# The Institutional Worker

**Published:** 1913-09-20

**Authors:** 


### INSTITUTIONAL WORKER

Being a Special Supplement to "The Hospital"

### OUR BUREAU OF INFORMATION.

### Rules for Correspondents.

1. Every letter must be accompanied by the coupon to be cut from the back cover (inside page) of The Hospital, current issue, and must contain the name and address of the correspondent with pseudonym for publication if desired. Replies by post cannot be given, save under exceptional circumstances at the Editor's discretion.

2. Letters from Approved Homes in reply to special needs published in the Bureau should state terms and full particulars, and be sent prepaid under cover to the Editor of the Bureau with name written across coupon for identification.

3. Proprietors of Homes which have not yet been entered on the List of Approved Homes, but have spare accommodation likely to suit special needs, are invited to write for an application form for registration. The fee for registration, which includes two announcements of the Home in the Bureau and other privileges, is 10s.

4. All communications to be addressed to the Editor of The Hospital, 28 Southampton Street, Strand, London, W.C., and marked "Bureau of Information."

### INSTITUTIONAL FACTS AND FIGURES.

### The Cost and Possibilities of Electrical Cooking.

It may perhaps surprise our readers, unless they studied with very great care the numerous debates that have been published so lavishly, to have their attention directed, as practical officials, to the engineering section of the British Association at Birmingham. Indeed, before considering the paper which Professor J. T. Morris contributed on the subject of electric heating and cooking, it is to be noticed that the least fruitful sections are always those which attempt to generalise, and the most useful, to those at least who intend to act upon what they learn, the sections which eschew principles for practical hints and detailed figures. Professor Smith was therefore following the most useful of precedents when he set out to give the results of one year's practical employment of electricity for cooking and heating purposes. He rightly began by pointing out that the question of cost was the prime factor, since the construction of the electric cooker had now been improved to the point at which, cost apart, it had quite justified its existence. Confining himself, therefore, to the economic aspect of cooking by electricity, he gave the units used each day for cooking over a period of ten months for half a dozen persons. As a result it was found that these varied from five to fifteen units, according, of course, to the kind and quantity of cooking required. The average daily consumption, however, worked out at 9.75 units, which at ½d. per unit amounted to 4.87d. per day, or about 2s. 9d. per week. The running cost, for supplying electrical energy both to the cooker and to the water for all household purposes, was rather less than 9d. per day.

Turning now to lighting, the cost worked out at 3½d. per week, or in other words at ½d. per unit 6.5 units were consumed. Including the cost of the telephone, the total annual expenditure worked out at £24 18s. 5d., and against this the Professor estimated that from £25 to £30 had been saved in the cost of domestic service, and thus the conclusion was drawn that on every ground electricity for domestic purposes had been justified.

#### The Need for Experiment.

We have preferred to set down these figures as they are without criticism, if only because an experiment of this kind must be tested for institutional purposes, by a direct institutional test. Experiments worked out by private enterprise (though highly valuable as showing what can be achieved by expert care when this care, too, is especially directed at proving the possibility of a new departure), are apt to break down when put to the less controlled and more rough-and-ready methods of practical institutional demands, where the human factor, inevitably under the best administration, counts for so much. For the most part, institutions have experimented so far rather with electrical devices than with electricity itself, and that is why the views of institutional officers are sometimes cloudy on new applications of electricity. For instance, only the other day we were shown in a ward kitchen an electrical device for warming plates, etc.; on observing that it was apparently disused, the explanation came as follows: This device, which was rather like a small gas oven in appearance, was a present to the institution, and the heating was given by three large electric globes. It was found in practice that these globes were always being broken through the carelessness of those who handled them and of course through their own brittleness. The cost of replacing them had proved so great that the stove was never used, and the present wasted.

#### A Suggestion.

Now the above is only a very rough-and-ready example of the sort of test, unexpected in a privately controlled experiment, which institutional improvements have to undergo. And it is knowledge of this kind which makes institutional workers very chary of theoretical demonstrations. However, the point in this case is that a series of figures has been published covering a reasonably long period, which may suggest to hospital men the lines on which some institutional experiment may be possible. That is why we record them here. The practical possibilities of electrical cooking have been demonstrated under certain conditions; can they also justify themselves under institutional conditions? What is the experience of hospital workers, so far as they have any of value, in this regard? It might be possible to test the claims here advanced on a small scale, for which the ward kitchen offers a suggestive field, provided that sufficient pains be taken to make a really careful record of the amount of units expended and the actual amount of work done and of cost incurred. We shall therefore be glad to hear from any of our readers who have observations to record or an opportunity for making them. Such observations, if published, would prove more suggestive than this paper, since they would have been made under the precise conditions which make the problem of utilising electricity for hospital purposes, apart from lighting, the peculiar and intricate thing which it is still.

### Questions for September.

No 1. Annual Cleaning.

What must be the essential details involved in the annual cleaning and painting, and what is the best method of supervising the work? [Detailed information as to the type and relative cost of paints and enamels found by experience to be most suitable should be given, and any suggestion on the best way to make the supervision effective will be favourably regarded in selecting the replies.]

No. 2. X-Ray Apparatus.

Give particulars of the X-ray apparatus in use at your institution, the names of makers and cost of installation. State the number of each class of cases dealt with, and give a detailed account of the annual cost of maintenance.

RULES.

The following rules must be observed:—

1. Contributions must be written on one side of the paper. They must bear the name and address of the sender and be accompanied by coupon to be cut from the back cover (inside page) of the current issue of The Hospital. A pseudonym must be chosen if the name is not to be published.

2. They must be addressed to the Editor of The Hospital, 28 & 29 Southampton Street, Strand, London, W.C., so as to reach him before the end of the month, and must be marked in the left-hand corner "Facts and Figures."

**A minimum payment of Half a Guinea will be made for each published answer.**

### *MISCELLANEOUS.*

**Gauge Glass Protector.**

The "Evertrusty" Gauge Glass Protector is one of the most efficient and reliable of the various types in the market, and can be obtained from Messrs. Wallach Bros., Ltd., of Finsbury Square, London, E.C. Made of clear white toughened glass, in which is embedded strong wire netting, it will resist almost any pressure or shock to which it may be subjected through the bursting of a gauge glass behind it. The "horse-shoe" pattern is generally used, and can be readily fixed and taken off. The price varies from 8s. 6d. to 13s. 6d., according to size. A "V" shaped protector is supplied by the same firm, which permits of the gauge glass being cleaned without removing the protector; this is somewhat more expensive.—L. P.

**"Duro" Fabrics.**

"Duro" fabrics, a sample of which you enclose, can be obtained of the British Textile Syndicate, 92 Market Street, Manchester.—"Uniform."

**We desire to inform numerous correspondents that we are unable to forward letters from or recommend any but our Approved Homes.**

### THE EDITOR'S LETTER-BOX.

### *EMPLOYMENT AND TRAINING.*

**Crippled Nurse.**

We have received your letter respecting the hospital nurse who has been an invalid for two years and is now only able to walk with the aid of crutches. We note that she is desirous of taking up office work, but has no means to enable her to obtain the necessary training. The case is a sad one, and can be placed before our readers as soon as you supply us with the references to which you allude and any further particulars that may be helpful. For instance, it would be helpful to us in dealing with the case to know where the nurse was trained, whether she is a member of the National Pension Fund, or any Nursing Association, and whether she would be willing to take up any work other than that suggested. —C. D. B.

**Holiday Research.**

A member of a hospital secretarial staff, who is taking an enforced holiday, asks if we can suggest any profitable way in which he can put his interest in hospitals to valuable use while not straining his health unduly. As he appears to incline to literature or research, we suggest that an inviting subject would be "Cots with a History." There are many, but the tales concerning them have never, we believe, been co-ordinated. If our correspondent cares to try we shall be very glad to consider his researches for publication. Can we say more?

O. H. B.

### *THE SICK AND IN NEED.*

**Home Wanted for Phthisical Case.**

We require a Home, as near Exeter as possible, for a young man who is suffering from phthisis in an advanced stage. A payment not exceeding a guinea a week could be made for his maintenance. Replies should be addressed C. S., 176.

**Home Wanted.**

A lady who has recently recovered from a long illness seeks a Home in an inland town in the South of England or close to London, where she could receive slight nursing care. She is in reduced circumstances and cannot afford payment exceeding 15s. a week. Replies should be addressed to "Cia," 173.

**Bath-chair Wanted.**

A poor woman who is a great sufferer from arthritic rheumatism is much in need of a bath-chair. We should be glad to hear from any of our readers able to offer one. References will be furnished, and a friend will gladly pay carriage. Replies should be addressed to J. W., 174.

**Lantern Slides.**

We do no know of any society or organisation likely to make you a free grant of lantern slides, but you could for a payment of 1s. a dozen obtain the loan of slides of the character you require for your lecture from the Women's Imperial Health Association, 7 Hanover Square, London, W.

### *ACKNOWLEDGMENTS.*

**L. S.**—We are gratified to hear that as a result of the suggestions made through our Bureau the poor man in whom you are interested is shortly to be received into a convalescent home.

**C. S.**—We have made it known in these columns that you require a Home for a young man who is suffering from advanced phthisis; meanwhile we suggest that you apply to St. Barnabas Home, Brocket Hall, Torquay.

**C. P. M.**—We are pleased to hear, in reference to your offer of a home and employment at your institution to a nurse unfitted for ordinary work, which we announced in these columns, that arrangements have been made with two of the candidates which will be mutually helpful.

**B. R.**—We are grateful to you for your letter and your kind promise to co-operate with us in providing for those in need of help.

**E. B.**—We are unable to introduce patients to any but Homes on our Approved List.

**E. J. Le Mar.**—We are glad to hear that you are happy and comfortable in your new quarters, and we much appreciate your kindness in forwarding us the cutting respecting the convalescent home.

*Communications have been received and attended to from L. Warburton, E. Coulter, M. McIlraith, L. Sherras.*

**The Editor is prepared to make known without charge the needs of any who may be sick or in difficulties, and to guide them in making choice of Homes for treatment or convalescence. Reduced terms can often be arranged with Homes on the Approved List for those unable to pay full fees. No Home is included in the Approved List without medical and other testimony to its good management. Queries are specially invited from those who are engaged in any kind of philanthropical work. A new edition of the leaflet describing the purposes of the Bureau of Information is now ready and will be sent free of charge on application. For Rules for Correspondents** **see page 1.**

### Editor's Notices.

**Contributions:**

Contributions should be written, or preferably typed, on one side of the paper only, and all articles sent in are accepted upon the distinct understanding that they are forwarded to THE HOSPITAL only.

The Editor cannot undertake to return MSS. not used, but when a stamped directed envelope is enclosed the MSS. may be returned if a special request is made.

Accepted articles and paragraphs of news will be paid for after publication at the scale rate.

**Address:**

To prevent delay all contributions and letters on editorial business must be addressed exclusively to the Editor, "The Hospital," 29 Southampton Street, Strand, London, W.C. It is important that this regulation shall be strictly observed.

**Correspondence:**

Correspondence on all subjects is invited. The name and address of correspondents must be given as a guarantee of good faith, but not necessarily for publication.

**Special Articles:**

Special articles are invited, and questions, inquiries, and paragraphs upon all matters relating to the work, administration and management of general, special, mental, fever, cottage and convalescent hospitals, sanatoria, homes, institutions, societies and organisations for the treatment or care of the sick, injured and dependants of all classes. Every matter affecting the interests and welfare of the staff of all grades working in these institutions will receive special consideration.

**Administrative Medicine, Research and Items of News:**

Special terms will be paid for approved articles on subjects in this wide field by experts who have made some section of it their special study and interest. We wish to give prominence to every movement tending to advance accuracy of diagnosis, efficiency in treatment, and the development of modern methods to eradicate disease and promote the welfare of the suffering.

**A Bureau of Information:**

We invite inquiries and applications for information and help in providing for that numerous class of sufferers who require special care or house-room which they cannot obtain in their own homes or secure for themselves. We cannot, however, prescribe, or recommend practitioners

**Books for Review:**

Publishers are particularly requested to send advance proofs of any new books of importance whenever possible, as the Editor has made arrangements to publish immediate reviews on a new plan.

**Photographs, Plans, Blocks, and Illustrations:**

It is requested that wherever possible MSS. may be accompanied by illustrations in any of the above forms. The name of the sender and of the article to which it belongs should be written on the back of each photograph, drawing, or block for purposes of identification.

**Local Papers:**

Newspapers containing reports or news paragraphs should be marked and addressed to the Sub-Editor.

**The Coupon System:**

A coupon will be found at the bottom of the third inside page of the cover each week. This coupon must be attached to every question or inquiry to which an answer is desired in THE HOSPITAL.

### When Seeking an Appointment.

As a means of announcing vacancies in every department of Institutional Work, the success of *The Institutional Worker* is evidenced by the ever-increasing number of advertisements that appear in this section of THE HOSPITAL. No more adequate proof of the value of *The Institutional Worker* can be desired than the fact that Authorities of the more important Institutions throughout the United Kingdom employ its columns again and again whenever they have a vacancy to fill upon their staffs. Moreover, it provides exceptional facilities for every class of announcement affecting Institutional Life and Work, and as a medium for securing appointments in any section of the Institutional, Health, and Sanitary Services it is a distinct help to the worker, since no other journal possesses such a comprehensive circulation in these directions as THE HOSPITAL. If you are seeking a post an announcement of twenty-one words, costing the small sum of 1s., will save you many hours of anxious thought, and certainly bring your requirements immediately to the notice of those most likely to need your services.

The form below may be used for Appointments Wanted advertisements, and when filled up should be posted to

*The Manager*, THE HOSPITAL,
28 and 29 Southampton Street,
Strand, W.C.

Appointments Wanted, 21 words ... 1s. 0d.

| | | |
|---|---|---|
| | | |
| | | |
| | | |
| | | |
| | | |
| | | |

### Manager's Notices.

**Letters relating to the publication, sale, and advertising departments of "The Hospital" must be addressed to The Manager, "The Hospital," The Hospital Building, 28 and 29 Southampton Street, London, W.C.**

**Advertisements:**

To ensure insertion, all advertisements for THE HOSPITAL must reach the Manager not later than Wednesday morning in each week. For scale of charges see pages v and 4.

**Subscriptions** may begin at any time, and are payable in advance. Cheques and Post-Office Orders should be crossed London County and Westminster Bank, Covent Garden Branch, and made payable to the Manager of THE HOSPITAL.

RATES OF SUBSCRIPTION (including postage).

| *United Kingdom.* | | *Foreign and Colonial.* | |
|---|---|---|---|
| Three Months ... ... | 2s. 0d | Three Months ... ... | 2s. 6d. |
| Six Months ... ... | 4s. 0d | Six Months ... ... | 5s. 0d. |
| Twelve Months ... ... | 6s. 6d | Twelve Months ... ... | 8s. 6d. |

**Remittances:**

**It is especially requested that remittances be made by Cheque or Postal Order, and in halfpenny stamps only when the amount is under sixpence.**

HANDBOOKS
For TRAINED WORKERS

POCKET-GUIDE SP SERIES

1/- net. 1/1 Post free.
Bound in Limp Cloth.
Suitable Size for the Pocket.

The works which comprise the S.P. Pocket Guide Series are intended for ready reference, consequently the aim of the Publishers has been to make them as concise and lucid as possible. Each book is written by an expert on the subject of which it treats, and the utmost reliance therefore can be placed in the information it imparts.

**Essentials of Fever Nursing.** By LYTTON MAITLAND, M.D. (Lond.), M.B., B.S., D.P.H. (Camb.).

**Manual and Atlas of Swedish Exercises.** *(With over 60 Illustrations.)* By THOMAS D. LUKE, M.D., F.R.C.S.

**Bandaging Made Easy.** *(Illustrated with over 90 Diagrams.)* By M. HOSKING, Sister-in-Charge, Tredegar House, Bow, E.

**How to Write and Read Prescriptions.** By LYTTON MAITLAND, M.D. (Lond.), M.B., B.S., D.P.H. (Camb.).

**Principal Drugs and their Uses.** By A PHARMACIST.

**Asepsis and How to Secure It.** By H. W. CARSON, F.R.C.S.

**Treatment after Operations.** *Rules for Nursing after General and Special Operations.* By MARY WILES.

**The Nurse's Duties before and during Operations.** By MARGARET FOX, Matron, Prince of Wales's General Hospital, Tottenham.

Other works, in amplification of this Series, will be announced in due course.

*Published by*
The SCIENTIFIC PRESS, Ltd.
28/29 Southampton Street, STRAND, LONDON, W.C.

